# Pilot Clinical Study Investigating the Thermal Physiology of Breast Cancer via High-Resolution Infrared Imaging

**DOI:** 10.3390/bioengineering8070086

**Published:** 2021-06-22

**Authors:** Adolfo Lozano, Jody C. Hayes, Lindsay M. Compton, Fatemeh Hassanipour

**Affiliations:** 1 Department of Mechanical Engineering, The University of Texas at Dallas, 800 W Campbell Rd, Mailstop ECW-31, Richardson, TX 75080, USA; adolfo.lozano@rtx.com; 2 Raytheon Intelligence & Space, 13510 North Central Expressway, Mailstop 212, Dallas, TX 75243, USA; 3 Department of Radiology, The University of Texas Southwestern Medical Center, 5323 Harry Hines Blvd. MC 8896, Dallas, TX 75390, USA; jody.hayes@utsouthwestern.edu (J.C.H.); lindsay.compton@utsouthwestern.edu (L.M.C.)

**Keywords:** infrared imaging, thermal imaging, breast cancer, hyperthermia

## Abstract

This descriptive study investigates breast thermal characteristics in females histologically diagnosed with unilateral breast cancer and in their contralateral normal breasts. The multi-institutional clinical pilot study was reviewed and approved by the Institutional Review Boards (IRBs) at participating institutions. Eleven female subjects with radiologic breast abnormalities were enrolled in the study between June 2019 and September 2019 after informed consent was obtained. Static infrared images were recorded for each subject. The Wilcoxon signed rank test was used to conduct paired comparisons in temperature data between breasts among the eight histologically diagnosed breast cancer subjects (*n* = 8). Localized temperatures of cancerous breast lesions were significantly warmer than corresponding regions in contralateral breasts (34.0 ± 0.9 °C vs. 33.2 ± 0.5 °C, *p* = 0.0142, 95% CI 0.25–1.5 °C). Generalized temperatures over cancerous breasts, in contrast, were not significantly warmer than corresponding regions in contralateral breasts (33.9 ± 0.8 °C vs. 33.4 ± 0.4 °C, *p* = 0.0625, 95% CI −0.05–1.45 °C). Among the breast cancers enrolled, breast cancers elevated temperatures locally at the site of the lesion (localized hyperthermia), but not over the entire breast (generalized hyperthermia).

## 1. Introduction

Breast cancer has been previously observed to exhibit elevated temperatures by varying amounts. While this pathophysiologic phenomenon has been the subject of numerous clinical investigations since the 1960s, these studies have largely investigated the screening and diagnostic efficacy of clinical thermal imaging. Whether some breast cancers are characterized by a measurable increase in surface temperature is not the subject of controversy [1]. In fact, numerous clinical studies have confirmed this hyperthermic phenomenon over the past six decades, as outlined in recent literature reviews [2,3,4]. Rather, it has historically been the use (or exploitation) of these increased temperatures as a screening or diagnostic criterion that has received significant medical scrutiny, owing to the mixed clinical findings, a lack of standardization in interpreting infrared (IR) breast images (also referred to as “thermograms”), and a lack of understanding of the fundamental thermophysiologic mechanisms of cancer.

The reason for which some breast cancers exhibit elevated temperatures is attributed to the established hallmarks of cancer that have only recently begun to be elucidated in the past decade [5]. The pathophysiologic changes associated with cancer can be classified into two categories: Metabolic changes and vascular changes, each of which are briefly summarized.

Altered cellular metabolism is associated with the sustained, chronic proliferation that is characteristic of cancer [6,7,8]. This altered energy metabolism, also known as the Warburg effect, refers to rapidly growing cells’ (e.g., cancer cells) preferred metabolic pathway for ATP production: aerobic glycolysis over mitochondrial oxidative phosphorylation. In effect, energy metabolism is reprogrammed in cancer cells to largely favor aerobic glycolysis, stopping short of oxidative phosphorylation, in order to take advantage of the production of the intermediate metabolites necessary to support rapid cellular growth and division. Interestingly, the glycolytic pathway is approximately 18 times less efficient in producing ATP molecules per glucose molecule than oxidative phosphorylation [9]. Cancer is therefore characterized by increased metabolic activity (i.e., an increased production of metabolites) as described by the two following hallmarks of cancer: excessive proliferative biochemical signaling and the 18-fold inefficiency in energy production [5,10,11].

Changes to the microvasculature are also associated with cancer [5,12,13]. These changes include the creation of new capillary blood vessels (angiogenesis) and the hijacking and vasodilation of existing nearby blood vessels (vascular co-option), all of which function to enhance blood flow in order to supply cancer cells’ increased demand for oxygen and nutrients [5,14,15,16,17]. Notably, Huang et al. recently observed this enhanced perfusion effect in brain tumors via blood oxygen level-dependent (BOLD) resting-state functional magnetic resonance imaging (rs-fMRI) [18].

Relatedly, inflammation is an enabling characteristic of cancer that is found in nearly all neoplastic lesions at varying densities [5]. Physiologically, inflammation occurs when host cells release biochemical signals upon injury or infection that induce vasodilation in nearby vessels, thereby locally increasing blood flow. Redness (erythema) and heat (calor) are typical symptoms of inflammation due to the increased perfusion [19]. In fact, with breast cancer specifically, inflammatory breast cancer is a phenotype characterized by gross inflammation that typically presents with palpable warmth as a symptom [20,21,22,23,24]. Although the inflammatory response is designed to repair or heal tissue, immune inflammatory cells counterintuitively have tumor-promoting characteristics that enhance angiogenesis and cellular proliferation [5].

Altogether, it is the aggregate effect of these thermophysiologic changes in cellular metabolism and the microvasculature which results in, at varying degrees, an increase in heat generation and bioheat transfer in cancerous tissue than in normal perfused tissue. Specifically, the excessive biochemical signaling and the increased production of metabolites associated with proliferation may be the primary source of metabolic heat generation in cancer, contributing to the heat generated by enhanced perfusion. In fact, a systematic investigation quantifying the degrees of hyperthermia according to breast cancer histologies, molecular subtypes, or lesion characteristics (e.g., size, location, grade, and stage) has never been conducted. These hallmarks and enabling characteristics of cancer have largely unexplored thermal implications [25].

The aim of this pilot study was to investigate the thermophysiologic characteristics of breast cancer by (1) collecting and analyzing high-resolution IR images and (2) constructing a computational thermal (or bioheat) model of the breast with and without cancer. Computational modeling results have been published previously [26]. Hence, this manuscript focuses on the clinical data collected during the study and provides a clinical assessment of breast cancer subjects’ temperature data in an effort to begin to understand the thermal physiology of breast cancer. The purpose of this study was not to assess the diagnostic accuracy of thermal (or infrared) imaging.

## 2. Materials and Methods

Female subjects (*n* = 11) at the Parkland Comprehensive Breast Center (Dallas, TX, USA) underwent standard, routine diagnostic exams (e.g., mammography, ultrasound, and magnetic resonance imaging). Subjects who were referred for breast biopsy on the basis of abnormal radiologic findings (specifically BIRADS 4B, 4C, and 5 diagnoses) were recruited for the unblinded study.

High-resolution static infrared images of 11 female subjects’ breasts were recorded. “High-resolution” herein refers to infrared camera pixel resolutions of 640 × 480 or greater, which is the higher end of resolutions commercially available [2]. The research imaging procedure took place prior to any treatment or surgical intervention, including breast biopsy, in order to preserve the original thermal characteristics of the potential cancer. Therefore, subjects’ histologic diagnoses were unknown at the time of IR imaging. Lesion definition (i.e., size and location) was determined by subjects’ standard radiologic imaging exams. Therefore, subjects’ radiologic imaging data were known and considered prior to IR imaging. In this study, IR imaging was not used to localize the internal cancerous lesion, but rather to thermally characterize the internal lesion (i.e., measure surface temperatures caused by the cancer).

### 2.1. Institutional Review Board Approval

This pilot clinical study was a collaborative, multi-institutional study between the University of Texas at Dallas, the University of Texas Southwestern Medical Center, and the Parkland Health & Hospital System. Accordingly, the study underwent review and approval by the following institutions: (1) the University of Texas Southwestern Medical Center’s Institutional Review Board (IRB Study No. STU-2018-0370), Breast Cancer Disease Oriented Team (DOT), and Protocol Review and Monitoring Committee (PRMC); (2) the University of Texas at Dallas’ Institutional Review Board (IRB Study No. 19-46); and (3) the Parkland Health & Hospital System’s Office of Research Administration (Study No. 27044). The clinical study commenced after receiving final approval from all institutions. The study was registered on www.ClinicalTrials.gov (accessed on 21 May 2019) (NCT03990012). Subjects were enrolled in the study after written informed consent was obtained. All research procedures were performed in accordance with relevant guidelines and regulations. Clinical data were collected between 13 June 2019 and 26 September 2019.

### 2.2. Procedure and Equipment

The IR imaging research procedure was non-invasive and posed minimal risk to subjects, without any exposure to harmful ionizing radiation. The IR imaging procedure lasted approximately 15 min per subject and took place immediately prior to each subject’s breast biopsy procedure.

High-resolution IR images were recorded using a FLIR A655sc IR camera (FLIR Systems Inc., Wilsonville, OR, USA). The IR camera had an uncooled microbolometer long-wave IR detector with a 640 × 480 resolution and 30 mK (0.030 °C) sensitivity (i.e., noise-equivalent temperature difference, or NETD). The standard IR camera lens was used (25° field of view, 24.6 mm focal length). Manufacturer datasheet is publicly available online.

The IR imaging procedure was generally based on that outlined by Ng [27]. Subjects entered the examination room and sat in the examination area. The examination room was dimly lit, free of air drafts, and did not have any windows. No major sources of heat (e.g., incandescent light bulbs) were located near the examination area. Subjects had been thermally acclimated to the environment by sitting for approximately 20 min immediately prior to the procedure (i.e., subjects were equilibrated to the ambient temperature). Subjects disrobed above the waist and the static IR images of subjects’ breasts under steady-state conditions were recorded from multiple angles in order to capture the full thermography of the breast.

After completing the IR imaging procedure, the 3D surface scanning of subjects’ breasts immediately followed. For the sake of brevity and scope, the 3D scanning procedure and data were previously outlined in [26]. After the 3D scanning procedure concluded, subjects re-dressed and went on to biopsy. Both research procedures combined lasted approximately 15 min per patient.

The ambient air temperature in the examination room was observed to range between 22.0 ${}^{{}^{\circ}}$C and 24.0 ${}^{{}^{\circ}}$C across imaging dates. Ambient temperature was measured using an Omega HH506RA digital thermometer (Omega Engineering Inc., Norwalk, CT, USA). The thermometer had a 0.1 ${}^{{}^{\circ}}$C resolution and ±0.3 ${}^{{}^{\circ}}$C accuracy with a manufacturer calibration certification to standard ANSI/Z540-1-1994. A T-type thermocouple of 36 AWG wire (0.13 mm diameter) with an exposed junction was used with the digital thermometer (part number 5SRTC-TT-T-36-36, Omega Engineering Inc., Norwalk, CT, USA). The thermocouple had a ±0.5 ${}^{{}^{\circ}}$C accuracy (“special limits of error” grade wire). The digital thermometer and thermocouple set’s accuracy was verified using a standard ice bath prior to usage, which resulted in a steady thermometer reading of 0.0 ${}^{{}^{\circ}}$C. Manufacturer datasheets are publicly available online.

Finally, subjects’ routine magnetic resonance (MR) imaging data were collected with an Optima MR450W GEM 70-CM 1.5T imager (General Electric Co., Florence, SC, USA) and provided the detailed internal data of the breast and lesion.

### 2.3. Data Analysis

IR images were analyzed using FLIR ResearchIR Max version 4.40.8.28, a vendor-provided proprietary software. Emissivity was set to a constant value of 0.98 as has been previously determined empirically for human skin [28,29,30].

For static IR images, two sets of temperature data were quantified: generalized and localized temperatures. Generalized temperatures were determined by drawing a region of interest (ROI) in FLIR ResearchIR Max over each subject’s two individual breasts as viewed on the frontal view IR images (hence “generalized”). In contrast, localized temperatures were determined by drawing an ROI over only the radiologically suspicious breast region (typically smaller in size) and a second ROI over the corresponding region in the contralateral normal breast. For each ROI, the mean temperature was considered. Extracting the localized temperatures typically required side view IR images. IR images with ROIs drawn for each subject are provided in the Supplementary Materials.

With regard to statistical methods, steady-state breast temperatures were analyzed on a per-breast basis from the histologically diagnosed breast cancer population (*n* = 8; Subjects 01–03, 05–06, 09–11). Two observations were made per subject among this population: normal breasts (*n* = 8) and cancerous breasts (*n* = 8), with the null hypothesis being that both breast groups exhibited the same temperatures (and therefore the same thermophysiologic characteristics). Here, the Wilcoxon signed rank test was used to conduct paired comparisons between the normal and cancerous breast groups for the generalized temperature set and localized temperature set (two tests). A *p* value of less than 0.05 was considered statistically significant in order to reject the null hypothesis. R version 4.0.0 with RStudio 1.2.5042 (RStudio PBC, Boston, MA, USA) was used for the statistical analysis. Resulting *p* values and 95% confidence intervals are reported. The three subjects whose histologic diagnoses were not breast cancer were excluded from the statistical population (Subjects 04, 07, and 08).

## 3. Results

### 3.1. Histologic Diagnoses

Subjects’ histologic diagnoses are presented in Table 1 for reference. Breast lesions were diagnosed as a malignant breast cancer in 8/11 subjects (Subjects 01–03, 05–06, 09-11), a benign breast disease in 2/11 subjects (Subjects 07–08), and a stromal cancer of non-mammary origin in 1/11 subjects (Subject 04). As noted previously, Subjects 04, 07, and 08 were excluded from the data analysis presented herein due to the subjects’ non-breast cancer diagnoses. All of the eight breast cancer subjects enrolled in the study presented with unilateral breast cancer (i.e., breast cancer in only one breast), as determined by their routine diagnostic imaging exams.

### 3.2. Static IR Images under Steady-State Conditions

**Figure 1:**
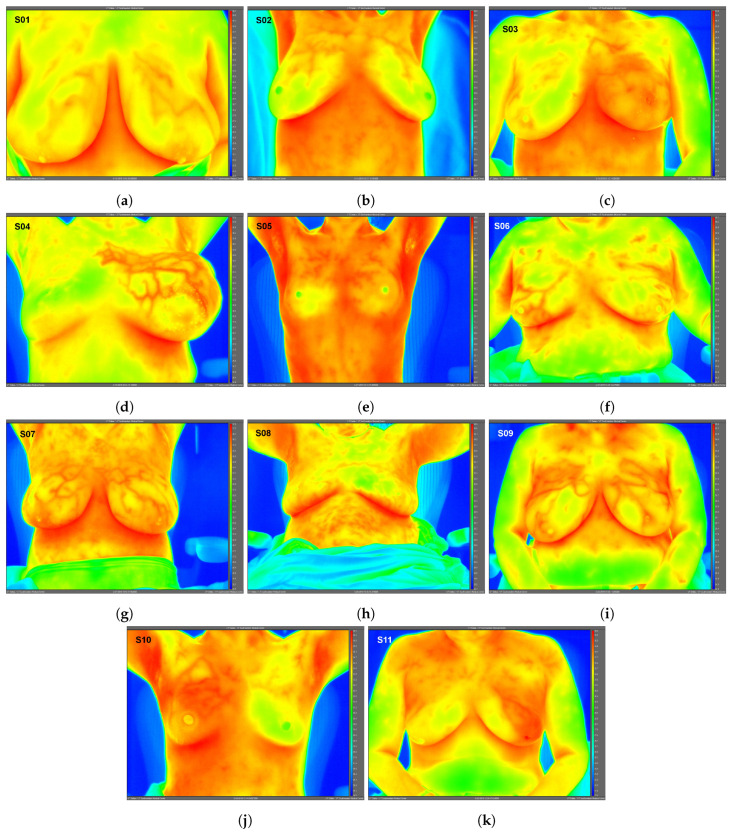
Static infrared (IR) images (frontal view) under steady-state conditions for Subjects 01 (**a**)–11 (**k**). Raw IR images with best image quality without any post-processing shown; therefore, subjects’ arms may be raised or lowered. Images exported using FLIR ResearchIR Max software. Note: Subject 08 (benign case, excluded from data analysis) inadvertently touched her neck and chest areas at least once immediately prior to IR imaging, altering surface temperatures and creating artificially low temperature regions. Image time stamps are in Coordinated Universal Time (UTC).

**Figure 2:**
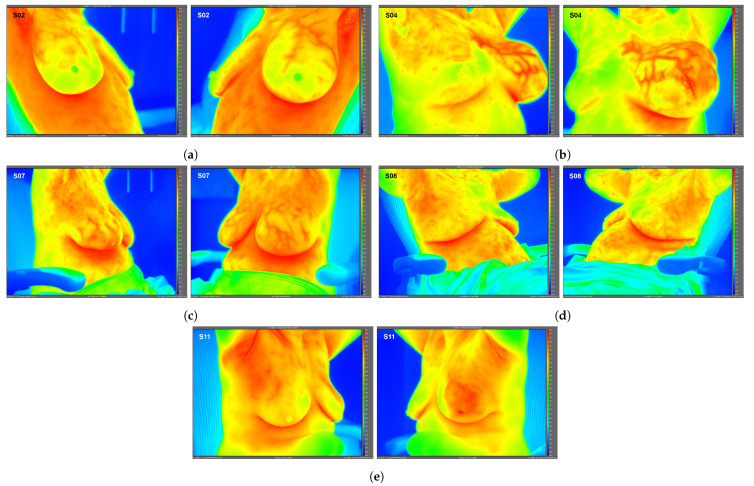
Side view static IR images for Subjects 02 (**a**), 04 (**b**), 07 (**c**), 08 (**d**), and 11 (**e**). Side view IR images were required for only these 5 subjects to quantify localized breast temperatures as presented in Table 2; frontal view IR images were used for all other subjects’ localized temperatures.

**Figure 3:**
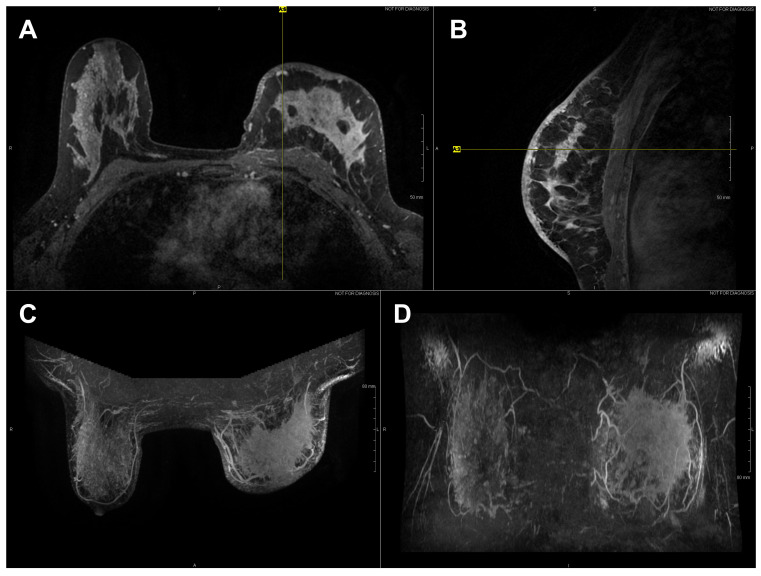
Axial (**A**) and sagittal (**B**) T1 contrast enhanced MR images of Subject 03’s malignant lesion. Axial (**C**) and coronal (**D**) maximum intensity projection images demonstrating large left breast mass and overlaying vasculature. Linear enhancing vessels on sagittal images appear as enhancing round or oval foci on axial cross-sections.

**Figure 4:**
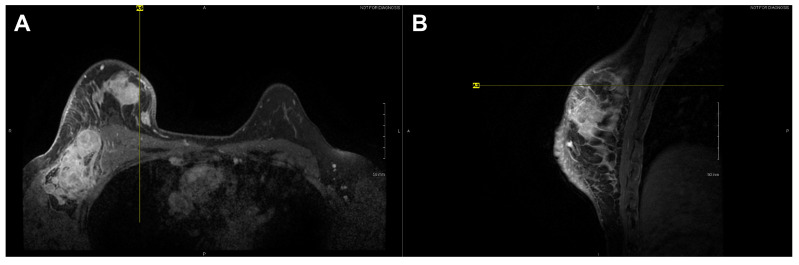
Axial (**A**) and sagittal (**B**) T1 contrast enhanced MR images of Subject 10’s malignant lesion. Linear enhancing vessels on sagittal images appear as enhancing round or oval foci on axial cross-sections.

High-resolution static IR images were collected from 11 female subjects with radiologic breast abnormalities under steady-state conditions. Subjects’ frontal view IR images are presented in Figure 1. Subjects’ side view IR images are presented in Figure 2. Steady-state mean temperatures based on static IR images are presented in Table 2. Finally, magnetic resonance imaging data are presented for the two subjects with the highest generalized temperatures observed: Subject 03 (35.0 ${}^{{}^{\circ}}$C, see Figure 3) and Subject 10 (35.3 ${}^{{}^{\circ}}$C, see Figure 4).

Among the 8/11 breast cancer subjects, generalized temperatures were on average 33.4 ± 0.4 ${}^{{}^{\circ}}$C for eight normal breasts and 33.9 ± 0.8 ${}^{{}^{\circ}}$C for eight cancerous breasts. No statistical significance was observed in generalized temperatures between normal breasts and cancerous breasts; the *p*-value and 95% confidence interval were: *p* = 0.0625, 95% CI −0.05–1.45 ${}^{{}^{\circ}}$C. In contrast, localized temperatures were on average 33.2 ± 0.5 ${}^{{}^{\circ}}$C for eight normal breasts and 34.0 ± 0.9 ${}^{{}^{\circ}}$C for eight cancerous breasts. Localized temperatures of breasts subsequently found to contain cancer were significantly warmer than corresponding regions on contralateral normal breasts. The *p*-value and 95% confidence interval were: *p* = 0.0142, 95% CI 0.25–1.5 ${}^{{}^{\circ}}$C.

To summarize statistical observations from steady-state temperature data, a significant difference in localized temperatures was observed between normal and cancerous breast temperatures among the eight histologically diagnosed breast cancers, but not in generalized temperatures.

## 4. Discussion

One advantage to high-resolution clinical IR imaging is that superficial thermovascular patterns on the skin are more easily observable than with lower resolution IR imaging, as observed by the inspection of Subjects 01, 04, 07, 09, and 11’s IR images (see Figure 1). The increased resolution is owed to the IR detector’s ability to resolve smaller temperature variations on the skin surface (i.e., lower noise-equivalent temperature difference, or NETD). Contrary to prior IR imaging studies with lower resolution IR equipment [32], recent clinical studies implementing high-resolution IR imaging more effectively revealed the surface vasculature of the breast by inspection of these studies’ IR images [33,34]. According to the hallmarks of cancer described in the Introduction, cancer induces changes to the local vasculature in order to support and sustain its rapid growth. Because blood serves as a heat source for resting adipose and glandular tissue such as the breast [35], the increased degree of perfusion will serve to elevate temperatures, as clearly observed in Subject 04 (see Figure 1).

The elevated localized temperatures observed among the eight breast cancers enrolled in this study (see Table 2) provide preliminary support for observations in the literature indicating that breast cancer can, in certain cases, lead to locally elevated tissue temperatures, as first observed by Lawson in 1956 [1,36]. In contrast, the lack of elevated generalized temperatures observed among the eight breast cancers suggested that not all breast cancers may sufficiently elevate the temperature of the entire cancerous breast to warrant the thermographic suspicion macroscopically as a screening procedure. Relevant factors include breast tissue composition, breast size, lesion size and depth as well as lesion characteristics (e.g., grade and stage). Given the diversity of breast cancers, more data across the various histologies and molecular subtypes of breast cancer will elucidate its effects on localized and generalized breast temperatures.

Subject 04’s clinical assessment warrants a separate discussion. Subject 04 presented with gross swelling and was histologically diagnosed with a malignant stromal cancer (Phyllodes tumor) in the subject’s left breast. Due to the non-mammary origin of the cancer (soft tissue sarcoma), this subject’s diagnosis was therefore not classified as a breast cancer (i.e., not of ductal or lobular origin). Swelling was the result of large volumes of cystic fluid (approximately several hundred cubic centimeters), which were drained during the biopsy procedure. As observed in Subject 04’s IR images, the aggressive stromal malignancy was characterized by excessive vascularization in order to support and sustain its growth; indeed, Phyllodes tumors are known to exhibit rapid growth rates [37].

Finally, the following were limitations to this pilot clinical study, which future clinical studies investigating breast cancer via high-resolution IR imaging should consider: (1) a small sample size of a breast cancer population was enrolled (*n* = 8). Enrolling more histologically diagnosed breast cancers may lead to a conclusive statistical finding regarding generalized temperatures between cancerous and normal breasts; and (2) subjects without any radiologic breast abnormalities (i.e., undiseased population) were not enrolled, which would have served as a control group for the unilateral breast cancer population.

## 5. Conclusions

This pilot clinical study of eight histologically diagnosed breast cancers found that breast cancer was associated with significantly elevated localized breast surface temperatures around subjects’ lesions, observable with static high-resolution IR imaging under steady-state conditions, but was not associated with significantly elevated generalized temperatures throughout subjects’ cancerous breasts.

High-resolution IR imaging is an essential tool in characterizing the thermal physiology of cancerous breast tissue. Although IR imaging presently serves a minor clinical role in the detection of breast cancer, it is nevertheless indispensable in the thermophysiologic characterization of normal and cancerous breast tissues. Understanding the thermal characteristics of breast cancer may support the implementation of thermal imaging in a clinical setting [26]. However, this first requires a systematic characterization of breast cancer in its full diversity with regard to histology, molecular subtype, grade, stage, size and location.

## Figures and Tables

**Table 1:** Summary of enrolled subjects and their corresponding histologic diagnoses. Subject Nos. with an asterisk (*) were excluded from the data analysis due to a non-breast cancer diagnosis. Lesion location is reported in standard BIRADS format: quadrant, clock-face position, distance (depth) from nipple. Abbreviations: “DCIS” ductal carcinoma in situ; “IDC” invasive ductal carcinoma; “IGM” idiopathic granulomatous mastitis; “LIQ” lower inner quadrant; “LOQ” lower outer quadrant; “mammo” mammography; “MR” magnetic resonance; “NOS” not otherwise specified; “UIQ” upper inner quadrant; “UOQ” upper outer quadrant; “US” ultrasound (i.e., sonography). None of the subjects enrolled were diagnosed with a lobular cancer, consistent with the prevalence rates reported by the American Cancer Society [31].

Subject No.	Diagnosis	Breast Side	Histology	Grade	Lesion Size	Lesion Location	Hormone Receptor Status	Stage	Notes
01	Malignant	Left	IDC	3	1.6 cm (US); 2.1 cm (MR)	UIQ, 11:00, 12 cm from nipple	ER−, PR−, HER2/neu−	1	Triple negative
02	Malignant	Left	DCIS	3	10 cm (mammo)	Calcifications centered at 3:00, spanning from 2 to 10 cm from nipple	ER−, PR−	0	Micropapillary
03	Malignant	Left	IDC (NOS)	2	10 cm (mammo)	UIQ, 11:00, 4 cm from nipple	ER−, PR−, HER2/neu−	4	Triple negative
04 *	Malignant	Left	Phyllodes	High	17.5 cm (mammo)	Mass occupied entire breast	–	–	Non-mammary origin (stromal cancer)
05	Malignant	Left	IDC	1	1 cm (US)	LIQ, 8:00, 3 cm from nipple	ER+, PR+, HER2/neu−	1a	–
06	Malignant	Right	IDC	3	1.8 cm (US); 2.8 cm (MR)	LIQ, 4:00, 6 cm from nipple	ER−, PR−, HER2/neu−	2b	Triple negative
07 *	Benign	Right	IGM	–	1.6 cm (US)	LOQ, 7:00, 6 cm from nipple	–	–	Pregnant (9 weeks)
08 *	Benign	Left	Fibroadenoma	–	1.9 cm (US)	UOQ, 2:00, 10 cm from nipple	–	–	–
09	Malignant	Left	IDC	3	3.6 cm (US)	12:00, 2 cm, from nipple (dominant mass) UOQ, 1:00, 2 cm from nipple (satellite mass)	ER+, PR+, HER2/neu+	2b	–
10	Malignant	Right	IDC	3	4.4 cm (US); 5.2 cm (MR)	UIQ, 1:00, 2 cm from nipple (dominant mass) UIQ, 2:00, 8 cm from nipple (satellite mass)	ER−, PR−, HER2/neu−	3b (inflammatory)	Triple negative
11	Malignant	Left	IDC (NOS)	3	8 cm (mammo)	UOQ, 2:00, 4 cm from nipple (dominant mass) UOQ, 2:00; UIQ, 10:00 (satellite masses)	ER−, PR−, HER2/neu−	2b	Triple negative; multiple adjacent satellite masses

**Table 2:** Static IR image temperature analysis summary. Generalized steady-state temperatures were obtained from a single frontal view IR image for each subject (presented in Figure 1). Localized steady-state temperatures were obtained from either frontal or side view IR images (presented in Figure 2). Regions of interest (ROIs) used are provided in the Supplementary Materials. “Normal” indicates that no radiologic abnormalities were observed from routine diagnostic breast exams.

Subject No.	Diagnosis	Side	Generalized Temperatures (${}^{{}^{\circ}}$C)	Localized Temperatures (${}^{{}^{\circ}}$C)
01	Breast cancer	Left	33.3	33.4
	Normal	Right	33.1	33.1
02	Breast cancer	Left	33.7	33.4
	Normal	Right	33.9	33.3
03	Breast cancer	Left	35.0	35.0
	Normal	Right	33.8	33.8
04	Breast cancer	Left	35.0	34.8
	Normal	Right	33.8	33.6
05	Breast cancer	Left	33.8	33.7
	Normal	Right	33.4	32.9
06	Breast cancer	Right	33.0	32.8
	Normal	Left	33.0	32.5
07	Breast cancer	Right	35.3	35.2
	Normal	Left	35.3	35.6
08	Breast cancer	Left	34.0	34.5
	Normal	Right	34.3	34.3
09	Breast cancer	Left	33.5	33.7
	Normal	Right	33.4	33.3
10	Breast cancer	Right	35.3	35.6
	Normal	Left	33.6	33.8
11	Breast cancer	Left	33.8	34.4
	Normal	Right	33.0	32.8

## Data Availability

All data generated or analyzed during this study are included in this published article (and its Supplementary Materials).

## Supplementary Materials

The following are available online at https://www.mdpi.com/article/10.3390/bioengineering8070086/s1: Figure S1: Static IR images indicating generalized regions of interest (ROIs) for Subjects 01–11; Figure S2: Static IR images indicating localized regions of interest (ROIs) for Subjects 01–11.

## Abbreviations

The following abbreviations are used in this manuscript:

CI	Confidence interval
BIRADS	Breast Imaging Reporting and Data System
DCIS	Ductal carcinoma in situ
IDC	Invasive ductal carcinoma
IGM	Idiopathic granulomatous mastitis
IR	Infrared
IRB	Institutional Review Board
LIQ	Lower inner quadrant
LOQ	Lower outer quadrant
MR	Magnetic resonance
NOS	Not otherwise specified
ROI	Region of interest
US	Ultrasound
UIQ	Upper inner quadrant
UOQ	Upper outer quadrant
