# Supplementary material for: Pilot Clinical Study Investigating the Thermal Physiology of Breast Cancer via High-Resolution Infrared Imaging
Source: Bioengineering (Basel). 2021 Jun 22;8(7):86. doi: 10.3390/bioengineering8070086 (PMC8301155; doi:10.3390/bioengineering8070086)
Supplement: Supplementary file 1 [file bioengineering-08-00086-s001.zip › bioengineering-1229429-supplementary.pdf]

## Supplementary Materials

Figure S1: Static IR images indicating generalized regions of interest (ROIs) for Subjects 01–11.
Figure S2: Static IR images indicating localized regions of interest (ROIs) for Subjects 01–11.

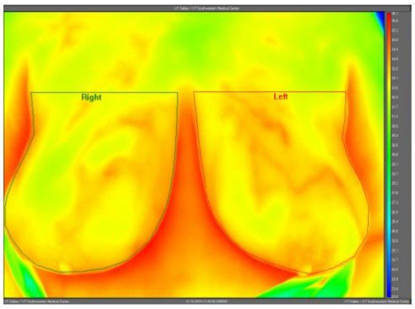

(**a**) Subject 01

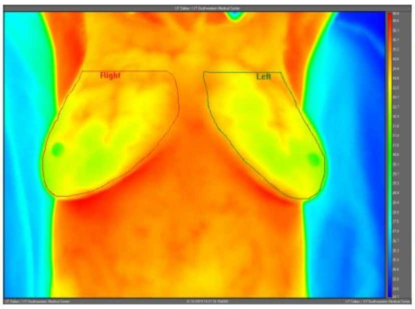

(**b**) Subject 02

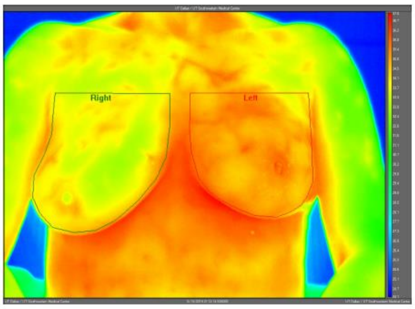

(**c**) Subject 03

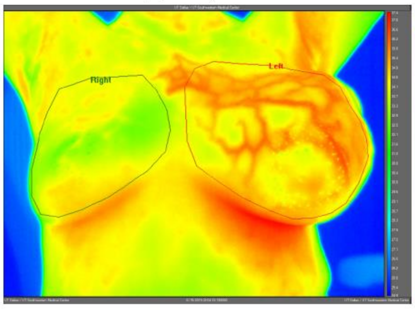

(**d**) Subject 04

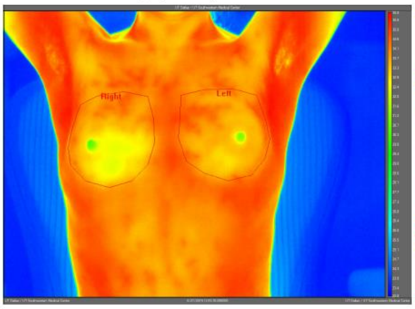

(**e**) Subject 05

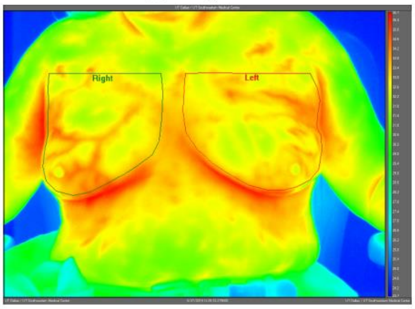

(**f**) Subject 06

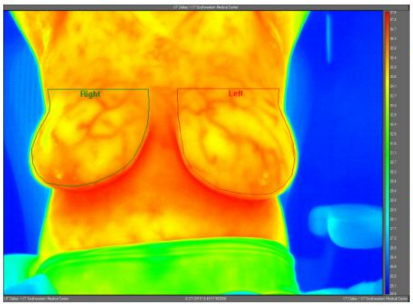

(**g**) Subject 07

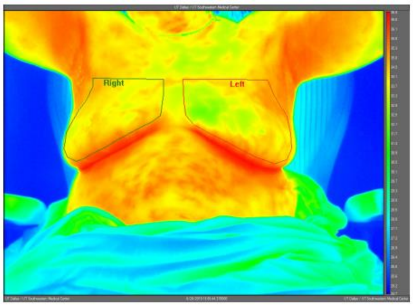

(**h**) Subject 08

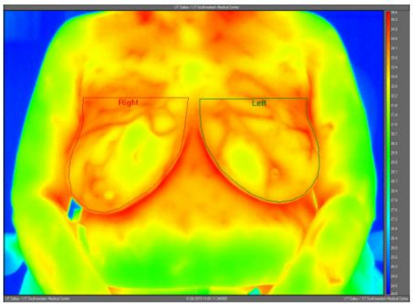

(**i**) Subject 09

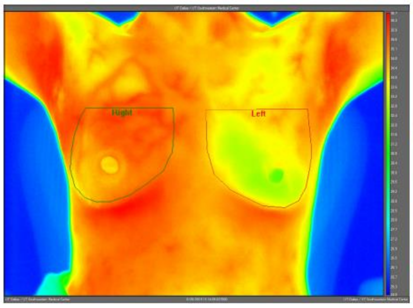

(**j**) Subject 10

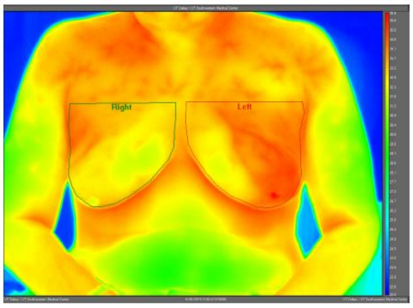

(**k**) Subject 11

**Figure S1.** Generalized regions of interest (ROIs) for Subjects 01–11 as outlined in Table 2. Generalized ROIs were drawn over the entire breast from subjects' frontal view IR images. Red and green ROI colors shown are arbitrary and have no significance.

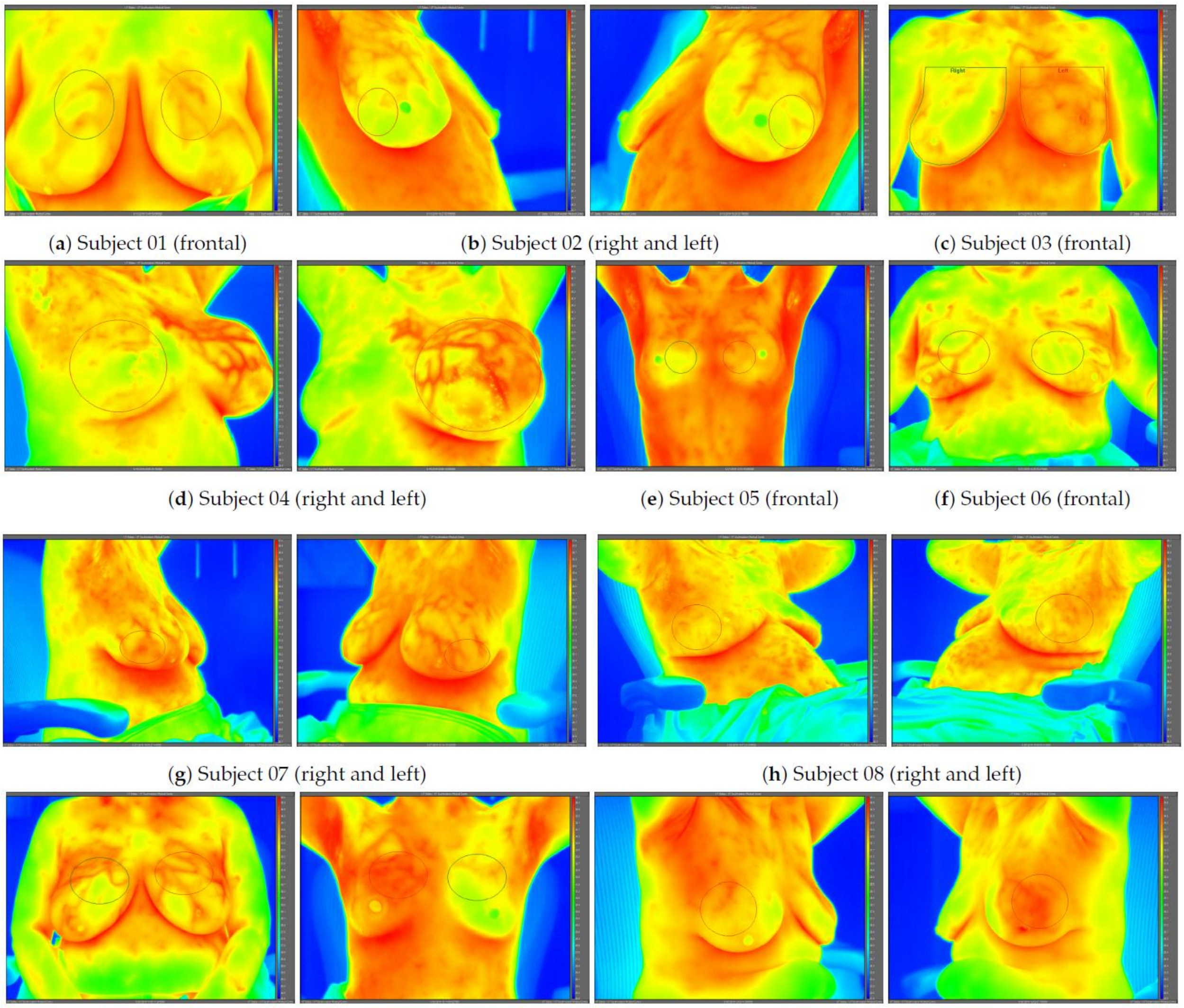

(**a**) Subject 01 (frontal) (**b**) Subject 02 (right and left) (**c**) Subject 03 (frontal)

(**d**) Subject 04 (right and left) (**e**) Subject 05 (frontal) (**f**) Subject 06 (frontal)

(**g**) Subject 07 (right and left) (**h**) Subject 08 (right and left)

(**i**) Subject 09 (frontal) (**j**) Subject 10 (frontal) (**k**) Subject 11 (right and left)

**Figure S2.** Localized regions of interest (ROIs) for Subjects 01–11 as outlined in Table 2. Localized ROIs were drawn around individual breast lesions as identified from subjects' radiologic imaging from subjects' frontal view or side view IR images (with the exception of Subject 03 whose localized ROI was identical to the generalized ROI because the lesion was widespread). Red and green ROI colors shown are arbitrary and have no significance.
